# Methadone Maintenance Treatment vs. Long-Term Abstinence Without Opioid Agonist: Epigenome-Wide Study of DNA Methylation

**DOI:** 10.3390/epigenomes10020029

**Published:** 2026-05-05

**Authors:** Orna Levran, Justin Li, Anat Sason, Miriam Adelson, Einat Peles

**Affiliations:** 1 Dr. Miriam & Sheldon G. Adelson Clinic for Drug Abuse Treatment & Research, Tel Aviv Sourasky University Medical Center, Tel Aviv 6423906, Israel; 2 The Laboratory of the Biology of Addictive Diseases, The Rockefeller University, New York, NY 10065, USA; 3 AccuraScience LLC, Johnston, IA 50131, USA; justin.t.li@lbs.accurascience.com; 4 Gray Faculty of Medical and Health Sciences, Tel Aviv University, Tel Aviv 6997801, Israel; 5 Sagol School of Neuroscience, Tel Aviv University, Tel Aviv 6997801, Israel

**Keywords:** epigenetic, DNA methylation, methadone, MMT, opioid use disorder, abstinence, cell adhesion

## Abstract

Background/Objectives: Opioid use disorder (OUD) is caused by a complex interplay between genetic and non-genetic factors. DNA methylation is an epigenetic mechanism that modulates gene expression. Data on DNA methylation and opioid addiction and treatment are limited. This association study was designed to assess the difference in genome-wide methylation patterns between individuals with OUD in methadone maintenance treatment (MMT) (*n* = 114) and those with OUD who achieved long-term abstinence (>10 years) without mu opioid receptor agonist treatment (*n* = 136). Methods: Differential DNA methylation analysis was performed in whole blood using the Illumina EPIC array. Results: A total of 135 differentially methylated probes (DMPs) reached epigenome-wide significance (*p* < 1 × 10^−7^), controlling for sex, age, estimates of blood cell proportions, and the first two principal components based on genome-wide SNP genotypes. The methylation sites were annotated to 157 genes, including 32% long non-coding RNAs. These genes are related to several systems, including cell adhesion (e.g., *SAXO4*), immune system and inflammation (e.g., *UBTF*, *USP39*, *C10orf90*, *PRKCA*), stress response (e.g., *CRHR1*, *GPR19*), and spermatogenesis (e.g., *SPATA16*, *COX7B2*). DMP cg11641410 is located in lncRNA *ENSG00000254687*, an antisense to *OPRK1*. Six of the DMPs were also identified in a related longitudinal study of MMT. Conclusions: At this point, it is not possible to determine whether the minor methylation differences observed in this study cause clinically relevant changes in gene expression. However, these findings have the potential to identify biomarkers and to provide new targets for treatment optimization.

## 1. Introduction

The development of opioid use disorder (OUD) involves a complex interplay between genetic and non-genetic factors. Individuals who try to abstain from heroin abuse face significant challenges and a high potential for relapse. Only a small proportion of individuals can maintain long-term abstinence without medication-assisted treatment. Methadone is a long-acting full opioid agonist, used as a cornerstone therapy in OUD, and is also an effective medication in the management of chronic pain. Methadone maintenance treatment (MMT) for OUD helps reduce withdrawal symptoms and cravings. Adequate methadone dose for patients in MMT is characterized by significant interindividual variability, relating to several factors including drug exposure, metabolism, and genetic variability. OUD is associated with systemic and central nervous system inflammation, and long-term methadone exposure may influence the immune system, potentially inducing or exacerbating the consequences of systemic inflammation [1,2]. Several studies suggested that MMT restores some immune functions [3]. Improved understanding of the response to methadone and long-term abstinence without medication is warranted.

Opioid abuse changes the brain reward circuitry, and these changes are mediated in part by epigenetic mechanisms. DNA methylation is an epigenetic mechanism that can influence DNA accessibility to regulatory proteins and modulate gene expression without changing the genetic sequence [4]. It is central to the interplay between genetic and non-genetic influences.

This study was designed to assess differences in genome-wide methylation patterns in blood DNA between subjects in long-term methadone maintenance treatment (MMT) and individuals with OUD who achieved long-term abstinence (>10 years) without mu opioid receptor agonist treatment (“medication-free”; MF) [5]. By comparing the two groups, we try to limit the effects of underlying predisposing genetic risk and past opioid abuse on the methylome. The differences between the two groups may reflect pharmacological effects of chronic methadone exposure, heroin-induced changes, polydrug use, comorbid conditions, response to stress, and lifestyle differences, among others.

Previous studies from our group suggested that MF individuals were able to update their associative learning, while MMT patients showed a selective impairment in reversing drug-related positive associations [6]. In addition, patients receiving MMT presented with a higher proportion of psychiatric comorbidity, and their perceived sleep quality and cognitive state were poorer than those of the MF individuals. MMT patients also suffered more chronic pain [5]. Benzodiazepine abuse is highly prevalent among MMT patients and was absent in the MF group. Two candidate gene studies of patients from these groups revealed an association between variants in stress-related genes and long-term abstinence, which may be related to stress resilience [7,8]. Verdejo et al. [9] reported more cognitive impairments in a group of MMT patients compared to a group of currently abstinent, former opioid abusers.

Data on DNA methylation and OUD are limited [10]. Candidate gene studies showed alterations in blood DNA methylation in the mu-opioid receptor gene (*OPRM1*) in individuals with heroin addiction (e.g., ref. [11]). Several epigenome-wide CpGs were identified in an association study of European American women with OUD [12] and in an Epigenome-wide association study (EWAS) of active injection drug use [13]. Additional related studies and preclinical studies can be found elsewhere (e.g., ref. [14,15,16,17,18]).

We hypothesize that individuals with ongoing MMT have distinct peripheral blood DNA methylation patterns compared to former opioid-dependent individuals in long-term abstinence. These patterns may serve as biomarkers and could provide mechanistic targets for treatment optimization.

## 2. Results

Differential DNA methylation analysis was performed in whole blood between opioid-dependent individuals in MMT (*n* = 114) and individuals in long-term abstinence (‘medication-free’; MF) (*n* = 136) using the Illumina EPIC array. After Bacon-based post hoc correction, a total of 135 DMPs reached epigenome-wide significance (*p* < 1 × 10^−7^), controlling for age, sex, batch, two population stratification PCs, and estimated compositions of six blood cell types (Supplement Table S1). DMPs with low methylation levels (normalized beta < 0.05) and DMPs with minimal differences in methylation levels between the groups (|delta beta| < 0.02) were excluded. DMPs reported to be related to tobacco smoking [19,20] were excluded.

**Figure 1:**
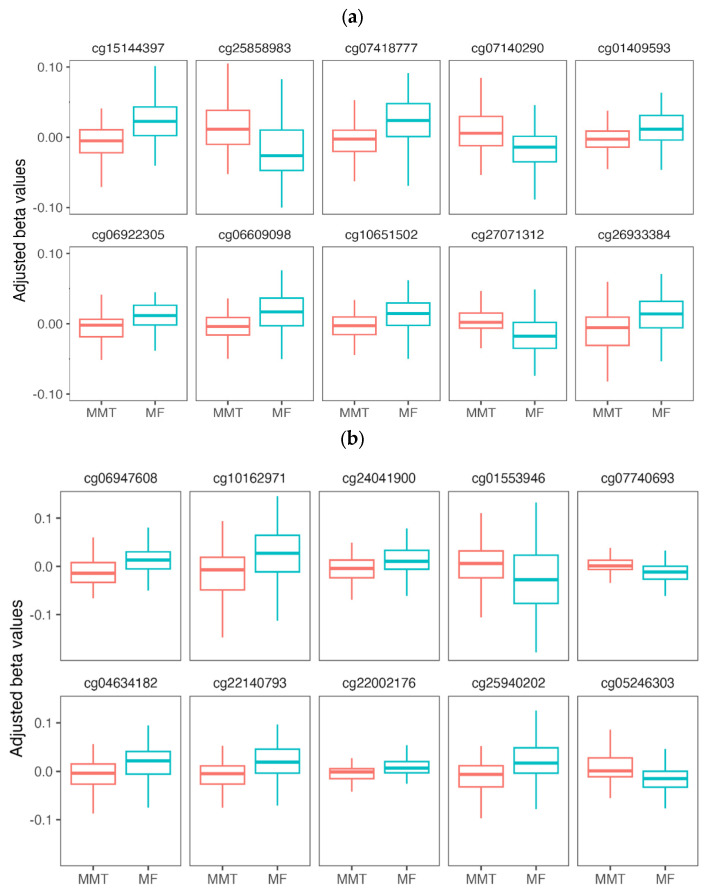
Methylation levels of the top-ranked DMPs in blood samples from subjects in long-term MMT and former opiate-dependent individuals in long-term abstinence without methadone treatment (MF). Adjusted beta values (*Y*-axis) represent the residual values after covariate adjustment: (**a**) DMPs 1–10. (**b**) DMPs 11–20.

The DMPs showed subtle differences in methylation levels (<4.3%). They are annotated to 157 genes, including 101 protein-coding (64%), 49 long non-coding RNAs (lncRNAs) (32%), and 7 other types (4%) (Supplement Table S2). A total of 45 DMPs are found within regulatory elements, including 11 DMPs in regions of promoter-like signature and 28 DMPs within CCCTC-binding factor (CTCF) bound regions (Supplement Table S1). Table 1 lists the characteristics and functional annotations of the 20 DMPs with the lowest adjusted *p*-values after Bacon correction. The residual beta means for each group and the differences in methylation levels between the groups (delta beta) are shown. Figure 1 shows the visual representation of the adjusted methylation levels (residual values after covariate adjustment) for these DMPs.

Box plots are defined as follows: the center line is the median. The box bounds show the interquartile range (IQR; 25th to 75th percentile). Whiskers extend to the smallest and largest values within 1.5 × IQR.

**Figure 2:**
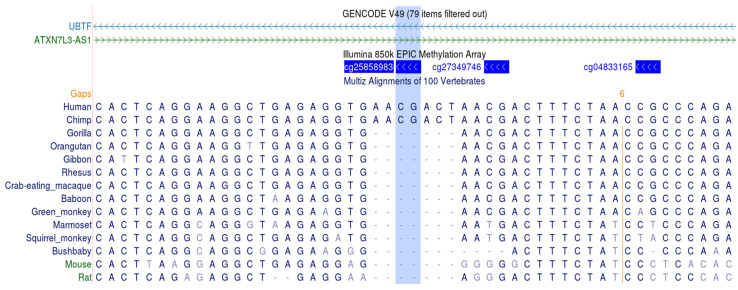
The genomic region of cg25858983 in *UBTF* and lncRNA *ATXN7L3-AS1*. Annotation data were extracted from the UCSC website. A 7 bp duplication that occurred in the common ancestor of humans, bonobos, and chimpanzees is shown in the bottom.

One of the top DMPs (cg25858983), annotated to the gene body of the upstream binding transcription factor gene, *UBTF*, and the lncRNA *ATXN7L3-AS1*, showed a 3.7% increase in methylation among MMT patients compared to the MF group (Table 1, Figure 1). This DMP is positioned within a CTCF-bound region with a proximal enhancer-like signature (Figure 2). Bioinformatic analysis revealed that it is located at a 7 bp duplication that occurred in the common ancestor of humans, bonobos, and chimpanzees, while the overlapping sequence is highly conserved.

DMP cg11641410, which is not on the top-ranked list, is found in an intron of lncRNA ENSG00000254687 (RP11-162D9.3) and is predominantly and highly expressed in testis cells. Notably, it is an antisense to the kappa-opioid receptor gene, *OPRK1*. In the current analysis, methylation levels in blood were high in both MMT and MF groups, with slightly higher levels in the MF group. An additional five genes on the annotated list are expressed in testis cells (*SPATA16*, *COX7B2*, and three lncRNAs). DMP cg04999148 is in a CTCF-bound region with a proximal enhancer-like signature upstream (TSS1500) of the lncRNA *NEAT1*.

It is not yet known whether the DMPs are correlated with gene expression or function. However, the annotated genes may be of interest. Several genes indicated in the current study are involved in the stress response, including *GPR19*, *CRHR1*, *GNA11*, *PRKCA*, and *MAP4K3*. DMP cg06928346 is in a CTCF-bound region with a promoter-like signature in the 5′ UTR of the G protein-coupled receptor 19 gene, *GPR19*.

DMP cg09422970 is in an intron of *CRHR1* encoding the corticotropin-releasing hormone receptor 1. The methylation levels of cg09422970 were high in both the MMT and MF groups, with slightly higher levels in the MF group. DMP cg04955246 is in a CTCF-bound intronic region with a distal enhancer-like signature in *PRKCA*. *PRKCA* encodes protein kinase C alpha, which plays a key role in opioid signaling, immune response, inflammation, stress response, cell adhesion, circadian rhythms, and pain modulation.

Several of the annotated genes are associated with GTPase-activator activity, including *ARHGAP15*, *HACD3*, *RIN3*, *SIPA1L2*, *TBCD*, and *TBCK*. A few of the annotated genes are related to the immune system, including *C10orf90 (FATS)*, *KREMEN2*, *RPS14*, *ITGA4*, and *UBTF*.

**Figure 3:**
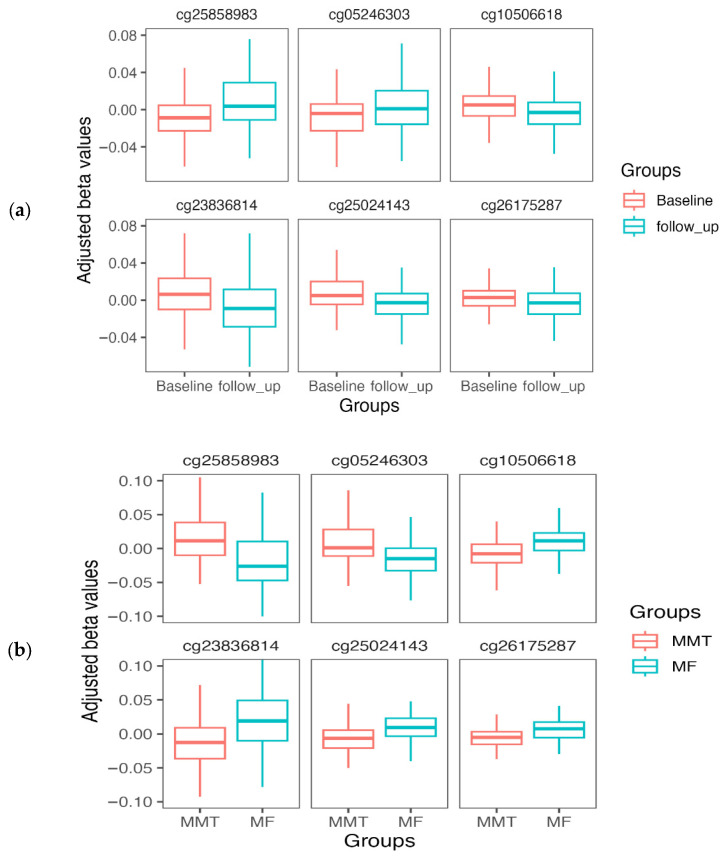
Adjusted methylation levels of DMPs that were identified in (**a**) a longitudinal study of MMT, as well as in (**b**) this study. Adjusted beta values (*Y*-axis) represent the residual values after covariate adjustment.

We have reported a longitudinal study of patients in MMT, using a subsample (*n* = 64) of the MMT group used in the current study [21]. Eight DMPs (annotated to 12 genes) from the current EWAS also showed significant differences in methylation levels after 1–3 years of MMT (Table 2), including two DMPs from the top 20 list. All these DMPs show a consistent effect when comparing the change during MMT to the difference between MF and MMT (Figure 3). DMP cg10506618 is located in an intronic region with an enhancer-like signature of *TRAK1*, which encodes the trafficking kinesin protein 1. The methylation level of the cg10506618 decreased from 58% to 52% after 1–3 years in MMT. In the EWAS, methylation levels were lower in MMT patients than in the MF sample.

## 3. Discussion

This study aimed to gain insight into the effect of MMT and long-term abstinence from opioid addiction, without medication, on DNA methylation. We compared the DNA methylome patterns in a group of MMT patients with those in a group in long-term abstinence using an EPIC-array-based EWAS in whole blood. Numerous factors, including predisposing risk, opioid abuse, methadone administration, and chronic inflammation, may determine variations in DNA methylation. By comparing these two groups, we aim to eliminate the effects of predisposing risk and earlier opioid addiction that are common to both groups.

A total of 135 DMPs reached epigenome-wide significance, with small differences in methylation levels. EWAS analyses of blood samples often show minor absolute differences in methylation levels between groups. One plausible reason is the mixture of different cell types in blood, which can dilute specific signals. Notably, 45 DMPs are found within regulatory elements and may change gene expression, but further studies are necessary to assess their functionality. While CpG methylation near a promoter generally represses gene transcription, CpG methylation within gene bodies has a more complex relationship with gene activity. Given the sample sizes, the observed variance of methylation beta values, and the epigenome-wide significance threshold, the study is sufficiently powered to detect small methylation differences on the order of approximately 2–3% delta beta. These DMPs can be biologically relevant and function as biomarkers.

Blood-based biomarkers are potentially important tools in the care of substance use disorder, and CpG methylation may provide a readout of gene environment interactions that influence treatment response. They could facilitate the development of epigenetic-based personalized therapeutic strategies.

### 3.1. Cell Adhesion Cytoskeletal Genes

One of the most significant DMPs is found upstream of *SAXO4* (aka *PPP1R32*; IIIG9; *C11orf66*). *SAXO4* is a regulatory subunit 32 of protein phosphatase 1 (PP1) that takes part in regulating motile cilia. *SAXO4* regulates the integrity of adherens junctions and is considered a gene of interest in the addiction field [22]. In addition, several genes related to GTPase activator activity were annotated by the identified DMPs, including *ARHGAP15*, *HACD3*, *RIN3*, *SIPA1L2*, *TBCD*, and *TBCK*. Rho GTPases regulate the cytoskeleton and cell–cell junction and interact with actin [23]. Actin filaments form the scaffold of the postsynaptic membrane. The cytoskeleton is vital to the integrity of brain activities, and it has the potential to modulate the plasticity associated with opioid abuse [24,25]. Another gene related to cell adhesion is *ITGA4*, which encodes the integrin subunit alpha 4. Cell adhesion molecules (CAMs) play an important role in neural plasticity, and several genes encoding CAMs have been associated with drug addiction [26]. CAMs also coordinate the transfer of immune components across the blood–brain barrier [27]. CAMs were suggested to be biomarkers for cytoskeletal dysfunction as well as opioid dependence [28].

An intriguing finding is the DMP cg04955246 in a regulatory and CTCF-bound region of *PRKCA*, which encodes protein kinase C alpha. Opioids primarily alter the function of G protein-coupled receptors, leading to activation of signaling pathways that in turn activate downstream protein kinases. This activation alters gene expression, which may contribute to addiction [29]. *PRKCA* plays a role in circadian rhythms, cell adhesion, synaptic plasticity, glutamatergic synapse, signal transduction, immune response, inflammation, and pain modulation.

### 3.2. Immune System and Inflammation

Several genes annotated by DMPs found in the current study are related to the immune system and to inflammation. USP39, which encodes ubiquitin-specific protease-39, regulates NF-kB-mediated inflammatory responses [30]. *RPS14*, encoding ribosomal protein S14, plays a role in the immune response [31,32]. *UBTF* encodes two isoforms of the upstream binding factor that play essential roles in ribosome biogenesis and innate antiviral immunity. Opioids may induce systemic inflammation in both the peripheral immune system and the brain [1,2,33]. Several studies suggested that MMT restores some immune functions [3]. Neuroinflammation induced by microglial activation can also impact stress-responsive pathways in the brain. Peripheral immune cells and peripherally released cytokines can infiltrate the brain and contribute to inflammation [34].

### 3.3. Stress and the Hypothalamic–Pituitary–Adrenal (HPA) Axis

Several of the genes annotated by the DMPs are related to the stress response, including *GPR19, CRHR1, GNA11*, *PRKCA*, and *MAP4K3*. Stress is one of the factors that may predispose individuals to substance use disorder [35]. The *CRH/CRHR1* system is a key regulator of the brain’s stress response. The *HPA* axis is regulated, in part, by endogenous opioids acting on mu-opioid receptors. DMP cg09422970 in *CRHR1* is intronic, and its function remains unknown. Potential support for the functionality of cg09422970 comes from a postmortem brain study that reported a difference in methylation levels of this CpG in subjects with AUD compared to controls [36]. GPR19 is an orphan G-protein-coupled receptor with a specific role in circadian regulation [37]. GPR19 coordinates multiple molecular aspects of stress responses associated with the aging process [38]. Although methylation profiles in blood do not directly reflect brain tissue, peripheral methylation changes can capture stress-related processes relevant to addiction.

### 3.4. Spermatogenesis-Associated Genes

*SPATA16*, indicated in this study, is associated with male infertility and globozoospermia [39]. Spermatogenesis is regulated in part by epigenetic factors, and abnormal spermatogenesis can result in male infertility. Opioids can cause alterations in the seminal RNA profile and reduce sperm production and quality [40,41]. Patients in MMT were found to have abnormal semen, but not all of them are necessarily pathological [42]. DNA methylation is tissue-specific, but some CpGs show cross-tissue correlation between testis and blood. These signals may be systemic epigenetic responses, as observed with methadone treatment, but must be confirmed in testicular cells.

### 3.5. lncRNAs

A total of 32% of the identified DMPs are annotated to lncRNAs. LncRNAs are non-coding transcripts of ≥200 nucleotides involved in several biological processes, including gene expression regulation. One of the indicated lncRNAs is *NEAT1*, which was found to be upregulated in the midbrain and NAc of heroin users [43]. *NEAT1* was reported to be upregulated by alcohol and nicotine and to affect neuronal plasticity [44]. DMP cg11641410 is annotated to lncRNA *ENSG00000254687*, which is predominantly and highly expressed in testis cells. Interestingly, it is an antisense to the kappa opioid receptor gene, *OPRK1*. It has the potential to alter the expression of *OPRK1*, which is associated with addiction, stress responses, and pain modulation. It may be a natural antisense transcript, but we could not find existing experimental data to support it. *OPRK1* is important for regulating inflammatory responses and is also expressed in the testis. In the current analysis, cg11641410 showed relatively high methylation in blood, with slightly higher methylation in the MF group. In support of the functionality of this CpG, data from a study of social anxiety disorder indicated low methylation of this CpG in blood from control samples compared to higher methylation levels in subjects with social anxiety disorder [20]. The high methylation of this CpG in both MMT and MF groups may be related to opioid addiction or stress levels.

### 3.6. Comparison to a Related Longitudinal Study

Eight DMPs identified in the current study were also reported in a related longitudinal study based on a subgroup of the current study’s MMT group [21]. All the DMPs showed a consistent effect on methylation levels when comparing changes during MMT to the difference between MF and MMT. A possible explanation for this effect is that the methylation change resulted from the steady methadone administration. Other explanations are possible, including life events, comorbidity, other substance use (e.g., benzodiazepine, cocaine), and other medications that were not accounted for due to power limitations.

### 3.7. Limitations

Several limitations should be considered. First, an EWAS is a cross-sectional study that cannot imply causation. Second, the absence of gene expression data prevents direct assessment of the functional consequences of the observed methylation differences. Third, DNA methylation is highly tissue-specific and may limit the interpretation of blood-based EWAS. Peripheral blood methylation may reflect changes in immune cell composition or systemic physiology relevant to opioid use disorder, but may not reflect relevant neural processes. Fourth, although this is a genome-wide study, the EPIC array only explores a small fraction of the human methylome. Fifth, there may be residual confounding (e.g., stressors, other drugs, comorbid conditions, and lifestyle), but the modest sample sizes limited the statistical power to account for them. Residual confounding would be expected to introduce variability rather than consistent directional effects, making the detection of significant CpGs more conservative.

### 3.8. Conclusions

This EWAS was designed to identify differences in genome-wide blood methylation patterns between MMT patients and individuals in long-term abstinence without mu-opioid receptor agonist treatment. A total of 135 DMPs, annotated to 157 genes, reached epigenome-wide significance. These potential genes of interest are related to various systems, including cell adhesion, immune system and inflammation, stress response, and spermatogenesis. At this point, it is not possible to determine whether the differences cause clinically relevant changes in gene expression. However, these findings have the potential to identify biomarkers and to provide new mechanistic targets for treatment optimization.

## 4. Materials and Methods

### 4.1. Study Samples

MMT patients (*n* = 114, 76% males, mean age = 48.2 years) were treated at the Dr. Miriam & Sheldon G. Adelson clinic for drug abuse treatment and research, Tel Aviv Sourasky Medical Center. All patients had a history of at least one year of daily multiple uses of opioids and at least two failures in a detoxification center (for details, see [45]). The patients in this sample have been treated with methadone for at least 3 months and no more than 3.5 years (an average treatment of 1.8 years). This subsample was collected for a longitudinal study that included patients under these specific criteria [21]. The Diagnostic and Statistical Manual of Mental Disorders, 4th Edition (DSM-IV), Kreek–McHugh–Schluger–Kellogg (KMSK) scale [46,47], and the Addiction Severity Index [48] were administered. Subjects with active DSM-IV Axis I disorder were excluded.

The medication-free (MF) sample (*n* = 136, 82% males, mean age = 50.9 years) included subjects who were previously treated in diverse addiction facilities, had several relapses, and maintained abstinence from heroin without medication treatment for at least 10 years [5]. Subjects who had a history of MMT for more than five months were excluded. All the subjects maintained abstinence from cocaine. Subjects were recruited at the Dr. Miriam & Sheldon G. Adelson clinic for drug abuse treatment and research, Tel Aviv Sourasky Medical Center. They had personal interviews that included the KMSK Scale and were tested for drugs of abuse in urine. A senior counselor from a different addiction treatment institute verified their histories.

Only subjects with a significant European and/or Middle Eastern descent based on self-report and principal component analysis (PCA) were included (see below). All the subjects signed informed consent for genetic studies. The study was approved by the institutional review boards of Tel Aviv Sourasky Medical Center (Helsinki Committee-03-240TLV).

### 4.2. Genome-Wide SNP Genotyping

DNA was extracted from peripheral blood using the Puregene Blood Kit (Qiagen, Germantown, MA, USA). DNA was quantified with the Qubit™ 3.0 fluorometer (Thermo Fisher Scientific, Waltham, MA, USA). The Illumina Infinium Global Screening Array (GSA-24 v3.0, Illumina, San Diego, CA, USA) was used for genotyping at the Icahn Institute for Genomics and Multiscale Biology at Mount Sinai (New York, NY, USA). The quality control inclusion criteria were: (1) detection of >99% at the individual level; (2) calling rate >99% at the SNP level; and (3) a *p*-value for the Hardy–Weinberg equilibrium > 1 × 10^−6^. Concordance with the EPIC array SNP probes was verified. PCA was conducted with PLINK (v1.9). Two PCs accounted for most of the variability in this sample and were included as covariates in the analyses.

### 4.3. DNA Methylation Profiling

DNA (1 microgram) was treated with a bisulfite conversion process using the Premium Bisulfite kit (Cat #C02030030) by Hologic Diagenode (Diagenode, Seraing, Belgium). The Illumina Infinium Human MethylationEPIC BeadChip array (v1.0 and v2.0) was used for DNA methylation (5-methylcytosine) quantification targeting >850,000 sites in biologically significant regions (Illumina, San Diego, CA, USA; Cat #G02090000, G0209006, respectively). Methylation levels were calculated as beta values, standing for the methylation percentage (range: 0–1).

### 4.4. Sample Quality Control (QC)

QC was performed based on internal control probes using GenomeStudio Software 2011.1, Methylation Module v1.9, as well as the *meffil* R package (v1.1.1) [49]. Samples with sex mismatch, detection *p*-value > 0.05, less than three beads per CpG, atypical blood cell-type composition estimates, and outlier signal in methylated vs. unmethylated comparison were excluded.

### 4.5. Probes Pre-Processing

Only probes that are included in the two versions of the EPIC array (v1.0 and v2.0, *n* = 721,745) were included. Probes mapped to sex chromosomes were excluded. A total of 705,485 probes were included in the analysis. Beta values were normalized using “meffil.normalize.samples”. A total of 671,001 probes were kept for analysis after excluding probes with normalized beta values below 0.05.

### 4.6. Estimated Blood Cell Subtype Proportions

Assessment of blood cell-type proportions was conducted using a multiplexed molecular platform based on CpG methylation data [50]. The relative proportions of six white blood cell types (i.e., B cells, T CD8+ cells, T CD4+ cells, natural killer cells, monocytes, and neutrophils) were estimated. A set of 306 CpGs (out of the original 329 CpGs [50]) was available after filtering. A linear model was constructed with sex, age, and batch (EPIC array version) as covariates. Adjusted beta values were obtained as the residuals from the linear model, accounting for covariate effects, and rescaled to the range 0 to 1. The function “meffil.estimate.cell.counts.from.betas”, which estimates cell counts from a reference, was used with the rescaled adjusted beta values. An optimized library for reference-based deconvolution was adopted as a reference using the function “blood IDOLoptimized epic” [51].

### 4.7. Epigenome-Wide Association Study (EWAS)

An EWAS analysis was conducted using the meffil R package and a regression model to compare methylation levels in blood samples from subjects in MMT and those who are medication-free. Age, sex, batch, two population stratification PCs, and estimated compositions of six blood cell types were included as independent variables. Adjusted beta values were obtained as residuals of the linear model. The significance threshold was set as *p* < 7 × 10^−7^. Genomic inflation was calculated using the “qq.inflation.method” parameter. Bacon-based post hoc correction was performed using the R package bacon (v1.32.0), with the summary statistics from the meffil-based EWAS analysis as input. False discovery rate (FDR) control was performed using the “p.adjust” function in R with the “method = BH” parameter. The threshold for significance was set as q-value < 0.05. Box plots were created using the “ggpubr” R package (version 0.6.0).

### 4.8. Bioinformatics Analysis

Annotation of differentially methylated positions (DMPs) was conducted using the annotation file of Illumina EPIC v2.0 resources. Gene annotations were performed using the current University of California, Santa Cruz UCSC RefGene annotation file (GENECODE v48). Genes were included if the distance between the CpG and the gene was less than 1.5 kb.

Additional information was obtained from Ensembl, the UCSC Genome Browser (http://genome.ucsc.edu/cgi-bin/hgGateway), the Genotype-Tissue Expression portal (GTEx) (https://gtexportal.org), the Human Protein Atlas (https://www.proteinatlas.org/), and the EWAS open platform (https://ngdc.cncb.ac.cn/ewas) [20]. Gene Ontology (GO) enrichment analysis was conducted using the clusterProfiler (version 4.10.0) package in R, including biological process (BP) pathways. Kyoto Encyclopedia of Genes and Genomes (KEGG) and Reactome pathway enrichment analyses were performed using the clusterProfiler (version 1.46.0) package in R.

## Figures and Tables

**Table 1:** Top-ranked 20 DMPs in the EWAS.

CpG	Chr	Map Info (hg38)	Adjusted *p*-Value (Bacon Correction)	Residual Beta Mean MMT	Residual Beta Mean MF	Delta Beta Residual	Nearest Genes	Gene Regions	Candidate Cis-Regulatory Elements (ENCODE)
cg15144397	3	172,911,234	1.56 × 10^−20^	−0.012	0.023	0.035	*SPATA16*	Intron	CTCF-bound
cg25858983	17	44,218,343	2.80 × 10^−19^	0.016	−0.021	−0.037	*UBTF* *ATXN7L3-AS1*	Exon; Intron	pELS; CTCF-bound
cg07418777	10	12,902,035	2.02 × 10^−17^	−0.011	0.021	0.032	*CCDC3 ENSG00000285520*	Intron	
cg07140290	16	2,964,185	3.60 × 10^−16^	0.011	−0.016	−0.027	*KREMEN2*	TSS200	PLS, CTCF-bound; CpG island
cg01409593	10	407,178	3.90 × 10^−15^	−0.007	0.013	0.020	*DIP2C*	Intron	dELS
cg06922305	2	85,615,376	1.55 × 10^−14^	−0.006	0.011	0.017	*USP39*	TSS1500; intron	
cg27071312	5	5,422,683	2.43 × 10^−14^	0.007	−0.015	−0.023	*ICE1* *ENSG00000286753*	5′ UTR; TSS1500	PLS, CTCF-bound; CpG island
cg06609098	11	61,480,963	1.61 × 10^−14^	−0.007	0.014	0.022	*SAXO4 ENSG00000256591*	TSS200; intron	
cg10651502	4	46,736,180	2.15 × 10^−14^	−0.006	0.011	0.018	*COX7B2*	Intron	
cg26933384	19	40,682,750	2.65 × 10^−13^	−0.010	0.013	0.023	*NUMBL*	coding sequence	
cg06947608	10	126,425,256	2.84 × 10^−13^	−0.012	0.012	0.023	*C10orf90 ENSG00000287326 LINC00601*	3′ UTR; TSS1500	dELS
cg10162971	2	9,953,364	3.22 × 10^−13^	−0.018	0.024	0.042	*GRHL1*	Intron	
cg24041900	8	143,742,461	3.28 × 10^−13^	−0.007	0.013	0.020	*IQANK1*	Intron	
cg01553946	5	150,449,373	5.51 × 10^−13^	0.012	−0.030	−0.042	*RPS14 ENSG00000295975*	5′ UTR; TSS1500	
cg07740693	6	41,371,878	5.97 × 10^−13^	0.005	−0.012	−0.017	*ENSG00000300220 ENSG00000300198*	Intron; exon; TSS200	dELS; CTCF-bound; CpG island
cg04634182	3	42,657,723	6.19 × 10^−13^	−0.009	0.019	0.028	*ZBTB47 ENSG00000300878*	Intron; TSS1500; TSS200	
cg22140793	8	94,274,517	6.51 × 10^−13^	−0.011	0.018	0.029	intergenic		
cg22002176	8	53,594,658	1.49 × 10^−12^	−0.005	0.008	0.013	intergenic		
cg25940202	1	207,811,449	2.13 × 10^−12^	−0.014	0.020	0.034	*MIR29B2CHG*	Intron	
cg05246303	2	181,456,627	4.29 × 10^−12^	0.011	−0.012	−0.023	*ENSG00000307562 ITGA4*	Exon; TSS1500	

dELS, distal enhancer-like signature; pELS, proximal enhancer-like signature; PLS, promoter-like signature; TSS1500, 1500 bp from the transcription start site; TSS200, 200 bp from the transcription start site.

**Table 2:** DMPs from this EWAS that were also identified in a related longitudinal study of MMT.

DMP	Adjusted *p*-Value	Adjusted *p*-Value (Bacon Correction)	*p*-Value Longitudinal Study	Nearest Genes
cg25858983	2.27 × 10^−28^	2.80 × 10^−19^	0.017	*UBTF*; *ATXN7L3-AS1*
cg05246303	1.43 × 10^−17^	4.29 × 10^−12^	0.049	*ITGA4*; *ENSG00000307562*
cg10506618	3.85 × 10^−17^	5.73 × 10^−12^	0.041	*TRAK1*
cg23836814	3.45 × 10^−16^	2.50 × 10^−11^	0.024	*AGBL2*
cg25024143	1.27 × 10^−14^	2.81 × 10^−10^	0.014	*SP2*; *SP2-AS1*
cg26175287	1.39 × 10^−13^	1.41 × 10^−9^	0.023	*SYNE3*

## Data Availability

Data are available upon request.

## Supplementary Materials

The following supporting information can be downloaded at https://www.mdpi.com/article/10.3390/epigenomes10020029/s1. Table S1 DMPs that reached epigenome-wide significance (*p* < 1 × 10^−7^) after Bacon-based post hoc correction; Table S2: Genes annotated by the significant DMPs.

## Abbreviations

The following abbreviations are used in this manuscript:

CAMs	Cell adhesion molecules
CTCF	CCCTC-binding factor
DMP	Differentially methylated probe
EWAS	Epigenome-wide association study
GO	Gene ontology
GTEx	Genotype-Tissue Expression portal
GSA	Global Screening Array
HPA	Hypothalamic–Pituitary–Adrenal
KMSK	Kreek–McHugh–Schluger–Kellogg
KEGG	Kyoto Encyclopedia of Genes and Genomes
lncRNA	Long non-coding RNA
MMT	Methadone maintenance treatment
OUD	Opioid use disorder
PCA	Principal component analysis
QC	Quality control
DSM-IV	The Diagnostic and Statistical Manual of Mental Disorders, 4th Edition
